# The pregnancy outcomes in patients with epididymal obstructive azoospermia after microsurgical vasoepididymostomy: a systematic review and meta-analysis

**DOI:** 10.3389/fmed.2023.1186729

**Published:** 2023-05-18

**Authors:** Zilong Wang, Xinkun Wang, Changze Song, Fuding Lu, Jiawen Zhai, Naifa Li, Baohong Jiang, Senbao Tan, Xujun Xuan

**Affiliations:** ^1^Department of Andrology, The Seventh Affiliated Hospital, Sun Yat-sen University, Shenzhen, China; ^2^National Research Center for Assisted Reproductive Technology and Reproductive Genetics, Cheeloo College of Medicine, Shandong University, Jinan, China

**Keywords:** microsurgical vasoepididymostomy, epididymal obstructive azoospermia, assisted reproductive technology, patency rate, natural pregnancy, pregnancy outcome

## Abstract

**Purpose:**

Pregnancy outcomes (overall patency rate, overall pregnancy rate, natural pregnancy rate, and the ratio of patients with pregnancy by assisted reproductive technology) after microsurgical vasoepididymostomy (MVE) in patients with epididymal obstructive azoospermia (EOA) were assessed through meta-analysis.

**Method:**

We searched PubMed, Embase, Web of Science, and the Cochrane Library databases up to 28 September 2022 for published literature related to retrospective or prospective clinical studies of obstructive azoospermia after apparent microsurgical vasoepididymostomy. Our search terms included obstructive azoospermia, epididymis obstruction, epididymal obstruction and vasoepididymostomy, and epididymovasostomy. Two researchers independently performed the literature search and assessed the eligibility of selected studies according to established inclusion criteria. The meta-analysis was performed using RevMan 5.4 software.

**Result:**

A total of 504 patients with EOA were included in 10 studies (including 2 prospective clinical studies and 8 retrospective clinical studies). The mean patency rate after MVE was 72% (95% *CI* 68–76%). The overall pregnancy rate was 34% (95% *CI* 30–38%). The natural pregnancy rate is 21% (95% *CI* 17–24%). The ratio of patients with pregnancy by assisted reproductive technology (ART) was 34.9%. For the factors affecting pregnancy outcomes after MVE, the overall pregnancy rates in patients receiving bilateral MVE were significantly higher than those receiving unilateral MVE (75.4 vs. 24.6%). The mean best sperm count and sperm motility in patients with overall pregnancy were significantly higher than those with failing pregnancies. For the subgroup meta-analysis of microsurgical vasoepididymostomy, there were no statistically significant differences in the overall patency rate (68 vs. 70%), the overall pregnancy rate (33 vs. 37%), the natural pregnancy rate (20 vs. 23%), the ratio of ART (30 vs. 28%) in end-to-side or end-to-end anastomosis, and longitudinal or triangular intussusception MVE.

**Conclusion:**

Vasectomy patency rates are higher, but natural pregnancy rates are lower in EOA male infertility patients after MVE. Altering the MVE procedures alone does not significantly improve pregnancy outcomes, but ART after MVE could improve the chance of pregnancy regardless of sperm parameters. We recommended that human sperms from EOA male infertility patients should be cryopreserved during intraoperative MVE for application in the subsequent ICSI treatment procedure.

## 1. Introduction

Obstructive azoospermia (OA) is the primary cause of male infertility, accounting for ~40% of male patients with azoospermia (1). Congenital or acquired OA is usually caused by the obstruction of the reproductive tract, including the vas deferens, epididymis, and ejaculatory duct. Among them, epididymal obstruction is the most common etiology of OA, and the prevalence of epididymal obstructive azoospermia (EOA) is 42.4–48% (2).

With the continuous improvement of assisted reproductive technologies (ART), reproductive tract reconstruction procedures and micro-testicular sperm extraction combined with intracytoplasmic sperm injection (ICSI) have become the main procedures of OA (3). However, as the previous gold standard for the treatment of male reproductive tract obstruction, microsurgical vasoepididymostomy (MVE) is also considered to be the procedure of choice for patients with EOA who are willing to achieve natural pregnancy (4). Although MVE can achieve natural pregnancy for patients with EOA, there are still some abnormal intraoperative complications, such as difficult genital tract separation or reconstruction (5), which are dependent on ART to achieve pregnancy (6). Therefore, the assessment of pregnancy outcomes is clinically important, including the overall patency rate, the overall pregnancy rate, the natural pregnancy rate, and the ratio of patients with pregnancy by ART.

The natural pregnancy rate of the MVE procedure is significantly lower than that of ICSI (7). However, the remediable treatment or secondary surgery after a failed MVE procedure increases the risk of surgical trauma and economic burden (8). Therefore, the purpose of this meta-analysis was to evaluate pregnancy outcomes in order to provide the best preoperative strategy for ART in patients with EOA after MVE.

## 2. Methods

### 2.1. Study retrieval

**Figure 1:**
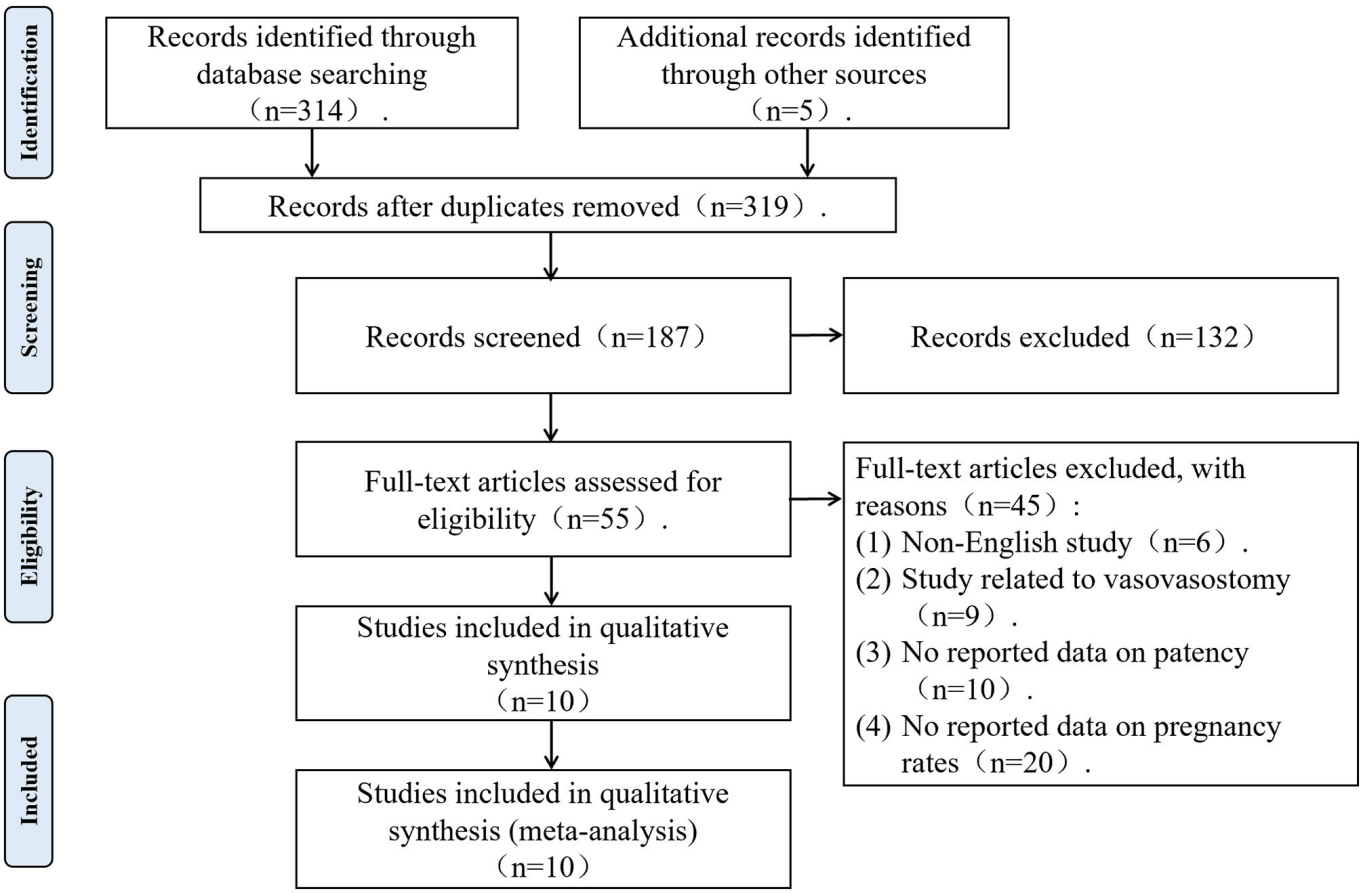
PRISMA flow diagram of the literature search and screening.

A literature search was performed following Preferred Reporting Items for Systematic Reviews and Meta-Analyses (PRISMA) Guidelines. We searched PubMed, EMBASE, Web of Science, and the Cochrane Library for English-language articles to collect prospective or retrospective clinical studies of apparent microsurgical vasoepididymostomy in patients with epididymal obstructive azoospermia from the start of the database to 28 September 2022. Search terms included “obstructive azoospermia,” “epididymis obstruction,” “epididymal obstruction” and “vasoepididymostomy,” and “epididymovasostomy.” In addition, all references of included studies were manually scanned to search for relevant articles in our study. The PRISMA flow diagram of the literature search and screening is shown in Figure 1.

### 2.2. Inclusion criterion

Study selection and eligibility screening were conducted according to the patient population, intervention or exposure, comparator, outcome, and study design (PICOS). All patients had a clinical examination and biopsy histopathological analysis confirming obstructive azoospermia (P). All of these patients received surgical treatment with microsurgical vasoepididymostomy (I). We compared two surgical procedures of microsurgical vasoepididymostomy, that is, end-to-side or end-to-end anastomosis and longitudinal or triangular intussusception for subgroup analysis (C). All clinical studies included pregnancy outcomes (the overall patency rate, the overall pregnancy rate, the natural pregnancy rate, and the ratio of assisted reproductive technology) (O). We included only retrospective or prospective cohort studies (S). The review articles and case reports were excluded from our study.

### 2.3. Data extraction

Two investigators (ZW and XW) independently conducted the screening and data extraction for the literature search. Articles were first screened based on titles and abstracts and then selected by full-text articles according to eligibility criteria. The following data were extracted for our study: authors, year, country, study type, quality assessment level, total numbers of cases, surgical procedures, and pregnancy outcomes (the overall patency rate, the overall pregnancy rate, the natural pregnancy rate, and the ratio of assisted reproductive technology).

### 2.4. Quality assessment

Two investigators (ZW and XW) independently completed the quality assessment and risk of bias for all included studies. We assessed the quality of single-group studies using the “Quality Assessment Tool for Before-After (Pre-Post) Studies with No Control Group Scale.” Publication bias was assessed by funnel plots and Begg's test.

### 2.5. Statistical analysis

Meta-analysis was performed in Review Manager (RevMan) version 5.4 to calculate the corresponding probabilities, standard errors, risk difference (RD), and 95% credible intervals (95% *CI*) as an effective index. After initial fixed-effects model analysis revealed a high degree of heterogeneity between studies (*I*^2^>50%) for subgroup analysis, a meta-analysis was performed using a fixed-effects model with a random-effects model. Publication bias was assessed using a funnel plot and Begg's test. The grouped analyses were performed in R language (3.6.1) software and statistical significance was determined with the *t*-test. A *P*-value of < 0.05 was considered to be statistically significant.

## 3. Results

### 3.1. Literature search process and results

The literature search yielded 319 potentially relevant articles, from which 10 relevant studies met the inclusion criteria (9–18), including eight prospective cohort studies (9–12, 14, 16–18) and two retrospective cohort studies (13, 15). The PRISMA flow diagram in Figure 1 shows the literature search process. These 10 relevant studies included a total of 504 patients who received MVE, of whom 348 achieved vasectomy patency. A total of 173 patients achieved pregnancies, of which 116 achieved natural pregnancy and 57 achieved pregnancy by ART (Table 1).

**Table 1:** Baseline characteristics of all included trials.

**Author**	**Year**	**Country**	**Study tape**	**QA grade**	**Case**	**Surgery methods**	**Patency rate**	**Pregnancy rate of spouses**	
								**Total**	**Natural**
Matsuda et al. (9)	1994	Japan	R	0.83	24	End-to-side/end anastomosis	80.8%	41.7%	37.5%
Jarow et al. (10)	1997	USA	R	0.83	131	End-to-side/end anastomosis	67.0%	27.5%	19.8%
Paick et al. (11)	2000	Korea	R	0.83	61	End-to-side/end anastomosis	68.9%	31.1%	18.0%
Hibi et al. (12)	2000	Japan	R	0.92	24	End-to-side/end anastomosis	54.2%	45.8%	16.7%
Chan et al. (13)	2005	USA	P	0.83	68	LIVE/TIVE	84.1%	51.5%	35.3%
Ho et al. (14)	2009	China	R	0.92	22	LIVE/TIVE	57.0%	31.8%	13.6%
Jiang et al. (15)	2014	China	P	0.92	51	LIVE/TIVE	82.4%	37.3%	31.3%
Binsaleh et al. (16)	2014	Saudi Arabia	R	0.92	22	LIVE/TIVE	59.1%	36.4%	13.6%
Zhao et al. (17)	2015	China	R	1.00	39	LIVE/TIVE	61.5%	38.5%	35.9%
Hong et al. (18)	2016	China	R	1.00	62	LIVE/TIVE	66.1%	22.6%	11.3%

QA, Quality Assessment; R, retrospective cohort study; P, prospective cohort study; LIVE, longitudinal intussusception vasoepididymostomy; TIVE, triangulation intussusception vasoepididymostomy.

### 3.2. The results of the meta-analysis

#### 3.2.1. The overall patency rate of the vas deferens after MVE

**Figure 2:**
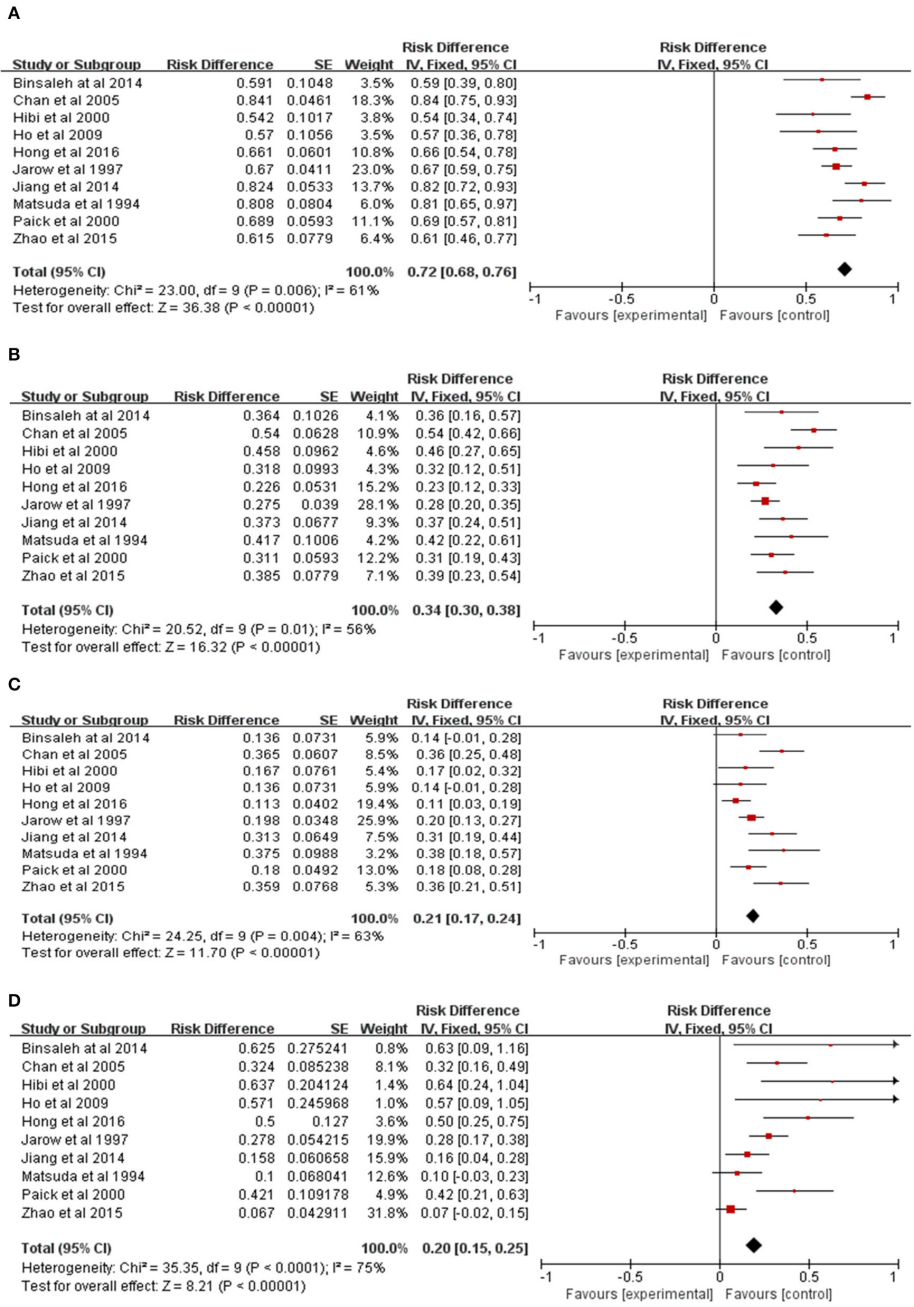
Meta-analysis of pregnancy outcomes in patients with EOA after MVE. **(A)** Meta-analysis of patency rate. **(B)** Meta-analysis of overall pregnancy rate. **(C)** Meta-analysis of natural pregnancy rate. **(D)** Meta-analysis of the ratio of pregnancy by ART.

A total of 10 clinical studies, including 504 patients were reviewed (9–18). In this analysis, a random-effect model was proposed for the high inter-study heterogeneity (*P* = 0.006, *I*^2^ = 61%). The results showed that the overall patency rate of the vas deferens after MVE was 72% (95% *CI* 68–76%, *P* < 0.0001, Figure 2A).

#### 3.2.2. The overall pregnancy rate after MVE

A total of 10 clinical studies, including 504 patients were reviewed (9–18). In this analysis, a random-effect model was presented for the high inter-study heterogeneity (*P* = 0.01, *I*^2^ = 56%). The results showed the overall pregnancy rate of 34% after MVE (95% *CI* 30–38%, *P* < 0.0001, Figure 2B).

#### 3.2.3. The natural pregnancy rate after MVE

A total of 10 clinical studies were reviewed for inclusion, including 504 patients (9–18). In this analysis, a random-effect model was presented for the high inter-study heterogeneity (*P* = 0.004, *I*^2^ = 63%). The results showed the natural pregnancy rate after MVE was 21% (95% *CI* 17–24%, *P* < 0.0001, Figure 2C).

#### 3.2.4. The ratio of pregnancy by ART after MVE

A total of 10 clinical studies were reviewed for inclusion, in which 173 patients' spouses achieved pregnancy (9–18). In this analysis, a random-effect model was presented for the high inter-study heterogeneity (*P* = 0.0002, *I*^2^ = 72%). There were 57 patients who achieved pregnancy by ART, including 26 ICSI cases, 21 *in vitro* fertilization and embryo transfer (IVF-ET) cases, and 10 intrauterine insemination (IUI) cases (Table 2). The results showed that the ratio of pregnancy by ART after MVE was 32.9% (Table 2; Figure 2D).

**Table 2:** Patients with pregnancy by ART of all included trials.

**Studies**	**Case**	**Overall pregnancy count**	**ART pregnancy count**	**The ratio of pregnancy by ART (%)**	**ART**		
					**ICSI**	**IVF-ET**	**IUI**
Matsuda et al. (9)	24	10	1	10	0	1	0
Jarow et al. (10)	131	36	10	27.8	1	2	7
Paick et al. (11)	61	19	8	42.1	0	8	0
Hibi et al. (12)	24	11	7	63.7	5	2	0
Chan et al. (13)	68	34	11	32.4	11	0	0
Ho et al. (14)	22	7	4	57.1	4	0	0
Jiang et al. (15)	51	19	3	15.8	0	0	3
Binsaleh et al. (16)	22	8	5	62.5	5	0	0
Zhao et al. (17)	39	15	1	6.7	0	1	0
Hong et al. (18)	62	14	7	50	0	7	0
**Total**	504	173	57	32.9	26	21	10

### 3.3. The factors affecting pregnancy outcomes after MVE

The factors affecting pregnancy outcomes after MVE includes the age of patients and their spouses, the etiology of obstruction, unilateral or bilateral operation, and postoperative sperm parameters.

#### 3.3.1. The ages of patients and their spouses

A total of 10 clinical studies reviewed for the ages of patients, including 504 patients, with a mean age of 35.12 (20–57) years (9–18). However, for the lack of statistical data on the ages of spouses in three studies (9, 10, 18), there are only seven clinical studies reviewed, including 287 patients, with a mean age of 28.89 (21–41) years (11–17) (Table 3). However, there is only one study showing the effect of a spouse's gestational age on pregnancy outcomes (17). The result showed that the mean age of the pregnant and non-pregnant spouses was 26.5 ± 4.5 (ranging from 21 to 34 years) and 32.7 ± 3.3 years old (ranging from 29 to 38 years) (*P* < 0.05).

**Table 3:** Ages and etiologies of all included trials.

**Studies**	**Case**	**Follow-up duration (months)**	**Age**		**Duration of obstruction (months)**		
			**Patients**	**Spouses**	**Pregnancy**	**Non-pregnancy**	**Etiology**
Matsuda et al. (9)	24	6–36	39	–	7.25 (1–15)	24.42 (17–34)	9 (37.5%) infection/epididymitis
							8 (33.3%) vasectomy/inguinal herniorrhaphy
							1 (4.2%) congenital
							6 (25%) idiopathic
Jarow et al. (10)	131	2–92	39	–	12–612		25 (19%) infection/epididymitis
							63 (48%) vasectomy
							26 (20%) congenital
							17 (13%) idiopathic
Paick et al. (11)	61	24–50	32	27	–	–	32 (52.5%) infection/epididymitis
							3 (4.9%) trauma
							26 (42.6%) idiopathic
Hibi et al. (12)	24	7–132	31	27	24–120		7 (29.17%) infection/epididymitis
							2 (8.33%) vasectomy/inguinal herniorrhaphy
							1 (4.17%) trauma
							14 (58.33%) idiopathic
Chan et al. (13)	68	1–36	40	32	18.8 (3–28)		15 (22.1%) infection/epididymitis
							34 (50%) vasectomy/inguinal herniorrhaphy
							1 (1.5%) trauma
							18 (26.4%) idiopathic
Ho et al. (14)	22	4–32	36	30	–	–	14 (63.6%) infection/epididymitis
							8 (36.4%) idiopathic
Jiang et al. (15)	51	9–52	32	29	–	–	13 (25.4%) infection/epididymitis
							3 (5.9%) vasectomy/inguinal herniorrhaphy
							35 (68.7%) idiopathic
Binsaleh et al. (16)	22	6–30	31	25	–	–	14 (63.6%) infection/epididymitis
							2 (9.1%) vasectomy/inguinal herniorrhaphy
							6 (27.3%) idiopathic
Zhao et al. (17)	39	2.5–12	31	29	36	32.4	14 (35.9%) infection/epididymitis
							25 (64.1%) idiopathic
Hong et al. (18)	62	6–12	31	–	–	–	16 (25.8%) infection/epididymitis
							46 (74.2%) idiopathic

#### 3.3.2. The etiology and duration of obstruction

The etiologies of obstruction mainly contain infection or epididymitis, vasectomy or inguinal herniorrhaphy, congenital anomalies, trauma, and idiopathic anomalies. A total of 10 clinical studies reviewed for these etiologies, including 504 patients (9–18). Overall, 159 (31.6%) patients suffered from infection or epididymitis, with 112 (22.2%) from vasectomy or inguinal herniorrhaphy, 27 (5.3%) from congenital anomalies, 5 (1%) from trauma, and 201 (39.9%) from idiopathic anomalies (Table 3).

Only five studies include the duration of obstruction (9, 10, 12, 13, 17) and only two above studies compared the duration between pregnancy and non-pregnancy (9, 17). However, for the lack of these patients' data from adolescence, the results showed no significant difference between pregnancy and the duration of obstruction (Table 3).

#### 3.3.3. Meta-analysis of unilateral or bilateral MVE

**Figure 3:**
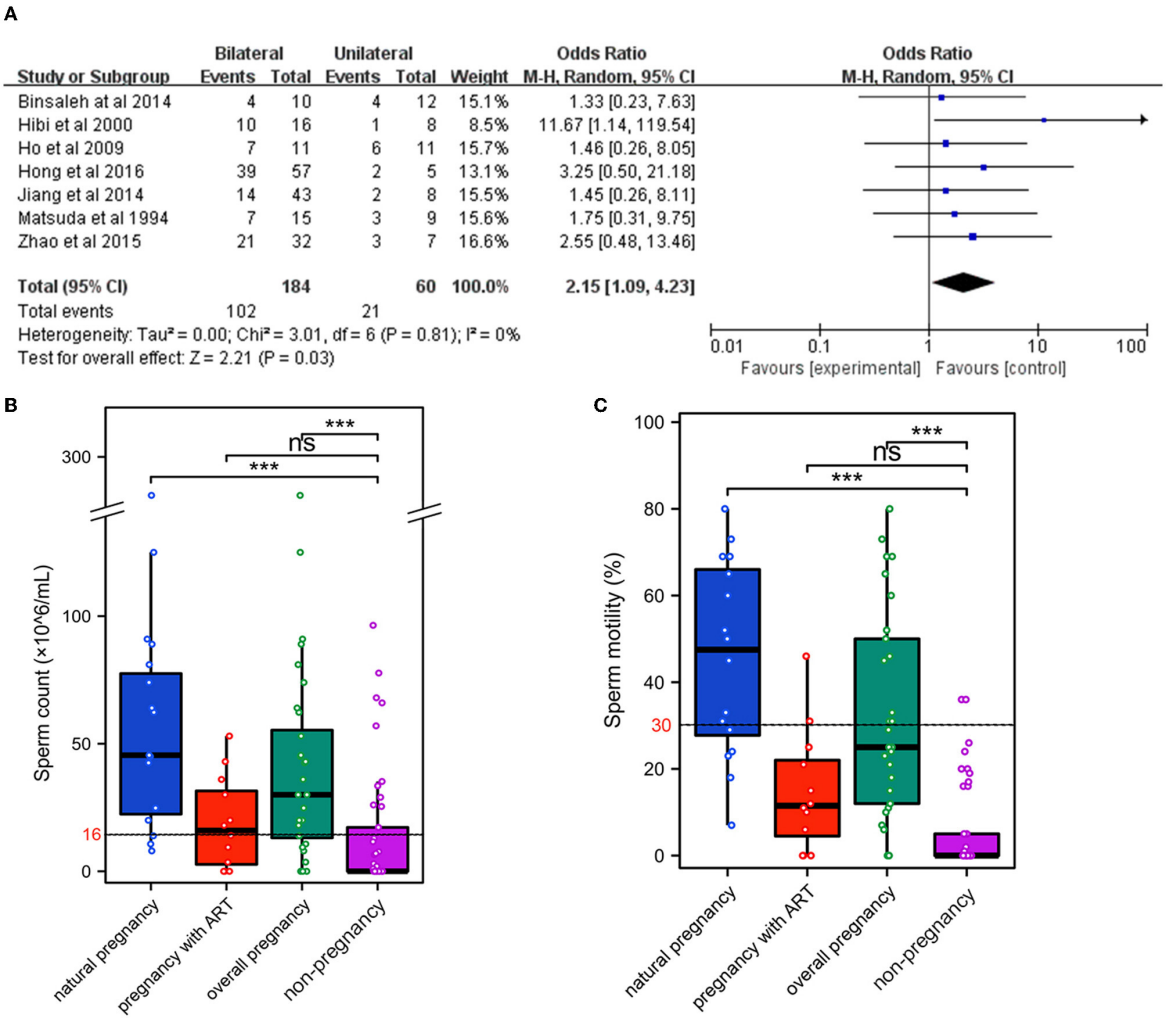
Factors affecting pregnancy outcomes after MVE. **(A)** Meta-analysis of unilateral or bilateral MVE. Grouped analyses of sperm parameters after MVE, including sperm count **(B)** and sperm motility **(C)**. ^***^P < 0.001.

A total of seven clinical studies, including 244 patients were reviewed (9, 12, 14–18). The overall pregnancy rates in patients receiving bilateral MVE were significantly higher than those receiving unilateral MVE (184 vs. 60, 75.4 vs. 24.6%, *P* = 0.03, Figure 3A; Table 4).

**Table 4:** Unilateral or bilateral MVE of all included trials.

**Studies**	**Case**	**Bilateral**			**Unilateral**		
		**Overall**	**Pregnancy**	**Non-pregnancy**	**Overall**	**Pregnancy**	**Non-pregnancy**
Matsuda et al. (9)	24	15	7	8	9	3	6
Hibi et al. (12)	24	16	10	6	8	1	7
Ho et al. (14)	22	11	7	4	11	6	5
Jiang et al. (15)	51	43	14	29	8	2	6
Binsaleh et al. (16)	22	10	4	6	12	4	8
Zhao et al. (17)	39	32	21	11	7	3	4
Hong et al. (18)	62	57	39	18	5	2	3
Total number	244	184	102	82	60	21	39
Total ratio (%)	–	75.4	41.8	33.6	24.6	8.6	16.0

#### 3.3.4. Grouped analyses and meta-analysis of sperm parameters after MVE

A total of six clinical studies contain sperm parameters after MVE (9, 12, 13, 16–18). These studies include 239 patients and the follow-up data containing sperm parameters were 219 patients (Table 5). The mean best sperm count was 21.8 × 10^6^/ml (normal range ≥ 16 × 10^6^/mL), and the mean best sperm motility (progressive motility rate, PR) was 19.4% (normal range ≥30%). The lower PR levels after MVE in semen samples could be the reason for the lower natural pregnancy rate.

**Table 5:** Sperm parameters after MVE of all included trials.

**Studies**	**Case**	**Semen count**	**Sperm count (**×**10**^**6**^**/mL)**					**Sperm motility (%)**				
			**Natural**	**ART**	**Pregnancy**	**Non-pregnancy**	**Mean**	**Natural**	**ART**	**Pregnancy**	**Non-pregnancy**	**Mean**
Matsuda et al. (9)	24	24	81.5 (24.8–267.7)	9.4	74.3 (9.4–267.7)	23.2 (0–96.4)	44.5 (0–267.7)	61.2 (33–80)	46	59.7 (33–80)	7.4 (0–36)	29.2 (0–80)
Hibi et al. (12)	24	24	25.5 (8–45)	5.4 (0–20)	15.5 (0–45)	1.0 (0–6.5)	7.7 (0–45)	25.5 (7–45)	11.2 (0–31)	17.6 (0–45)	2.9 (0–20)	9.6 (0–45)
Chan et al. (13)	68	63	–	–	–	–	12.8 (0.01–80)	–	–	–	–	21.0 (0–30)
Binsaleh et al. (16)	22	22	81.3 (74–89)	32.8 (14–53)	51.0 (14–89)	17.6 (0–68)	29.7 (0–89)	45.3 (27–57)	28.6 (15–44)	34.9 (15–57)	16.2 (0–51)	11.0 (0–57)
Zhao et al. (17)	39	24	–	–	49.1	36.0	42.1 (0.7–103)	–	–	15.1	6.7	10.9 (0–28)
Hong et al. (18)	62	62	–	–	–	–	17.1 (0–51.8)	–	–	–	–	24.0 (0–52)
**Average**			66.5	18.9	46.8	22.3	21.8	45.5	14.8	26.28	6.2	19.4
**Total**	239	219										

There are three clinical studies including all sperm parameters of every patient (9, 12, 16). We performed grouped analyses for sperm count and sperm motility of these 70 patients. The mean best sperm count in patients with natural pregnancy and overall pregnancy were significantly higher than those failing pregnancy, respectively (66.5 × 10^6^/ml *vs*. 22.3 × 10^6^/ml, 46.8 × 10^6^/ml *vs*. 22.3 × 10^6^/ml, *P* < 0.001, Figure 3B). The same results showed the mean best PR levels in these patients (45.5 vs. 6.2%, 36.3 vs. 6.2%, *P* < 0.001, Figure 3C). However, the mean best sperm count and PR levels in patients with pregnancy by ART were not significantly higher (Figures 3B, C). The mean best sperm count in these patients (18.9 × 10^6^/mL) was slightly higher than the normal ranges, and the PR levels (14.8%) were even lower than the normal ranges (Table 5).

We also performed continuous meta-analysis for sperm count and motility in these three clinical studies (9, 12, 16). The sperm count and motility in patients with overall pregnancy were significantly higher than those failing to achieve pregnancy, respectively (Supplementary Figures 1A, B), but those in natural pregnancy were not significantly higher (Supplementary Figures 1C, D). Furthermore, there were no significant differences between the sperm count or motility and successful pregnancy by ART after MVE (Supplementary Figures 1E, F). Therefore, combined with the data on the overall pregnancy rate and the ratio of patients with pregnancy by ART, ART after MVE could improve the chance of pregnancy regardless of sperm parameters.

### 3.4. The results of the subgroup analysis

#### 3.4.1. The overall patency rate of the vas deferens in different surgical procedures

**Figure 4:**
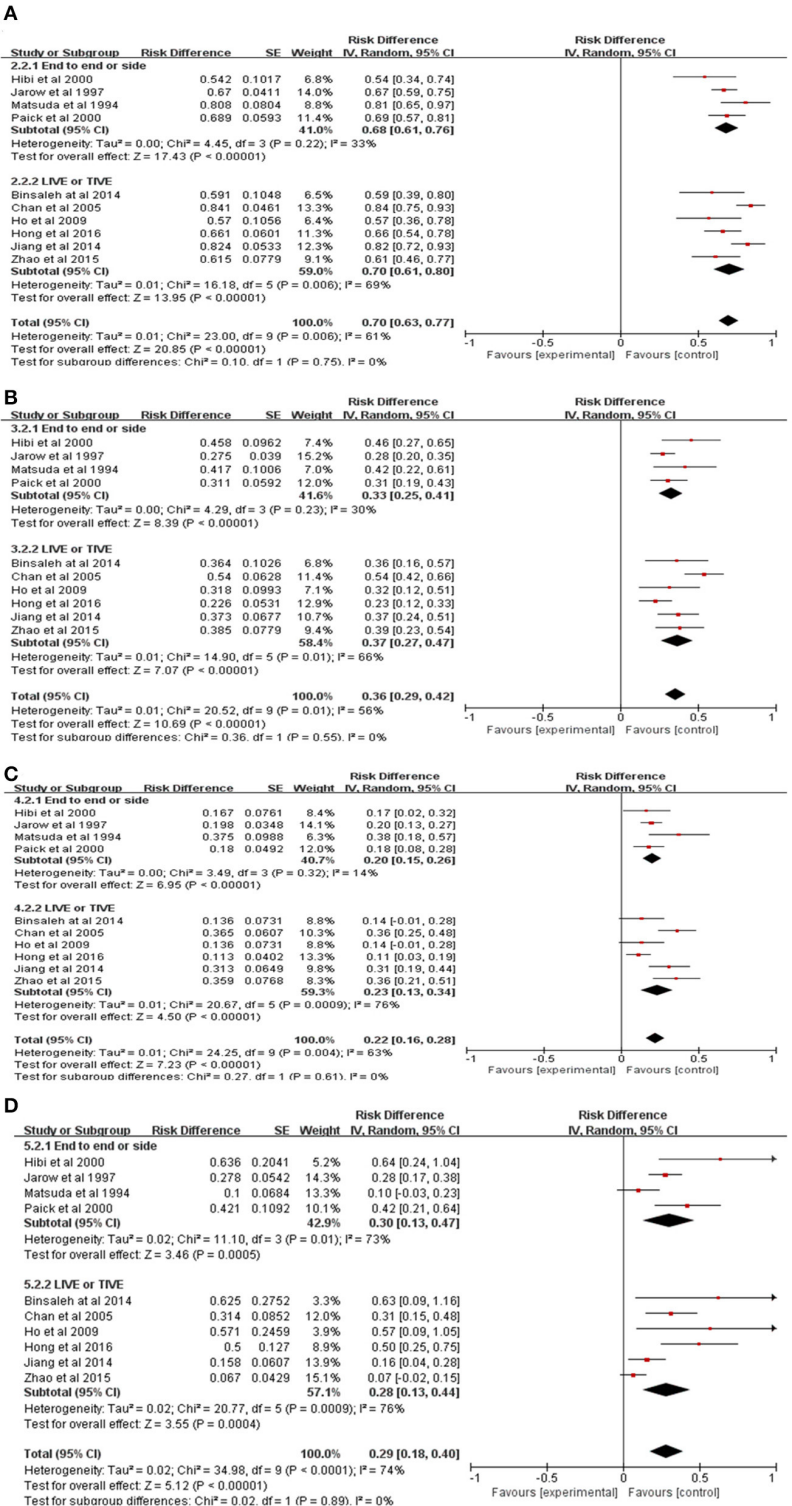
Subgroup meta-analysis of pregnancy outcomes in patients with EOA after MVE. **(A)** Subgroup meta-analysis of patency rate. **(B)** Subgroup meta-analysis of overall pregnancy rate. **(C)** Subgroup meta-analysis of natural pregnancy rate. **(D)** Subgroup meta-analysis of the ratio of pregnancy by ART.

A total of four clinical studies with end-to-side or end-to-end anastomosis (9–12) and six clinical studies with longitudinal or triangular intussusception (13–18) were included in the subgroup analysis. In this subgroup analysis, the random-effect models were uniformly presented as a high inter-study heterogeneity (*P* = 0.22 *vs*. 0.006, *I*^2^ = 33 vs. 69%, respectively). The results showed that the overall patency rates of the vas deferens after end-to-side or end-to-end anastomosis and longitudinal or triangular intussusception were 68% and 70% (95% *CI* 61–76 vs. 61–80%, *P* < 0.0001 *vs. P* < 0.0001, Figure 4A), respectively.

#### 3.4.2. The overall pregnancy rate in different surgical procedures

A total of four clinical studies with end-to-side or end-to-end anastomosis (9–12) and six clinical studies with longitudinal or triangular intussusception (13–18) were included in the subgroup analysis. In this subgroup analysis, the random-effect models were uniformly presented as a high inter-study heterogeneity (*P* = 0.23 *vs*. 0.01, *I*^2^ = 30 vs. 66%, respectively). The results showed that the overall pregnancy rates for the vas deferens after end-to-side or end-to-end anastomosis and longitudinal or triangular intussusception were 33% and 37% (95% *CI* 25–41 vs. 27–47%, *P* < 0.0001 *vs. P* < 0.0001, Figure 4B), respectively.

#### 3.4.3. The natural pregnancy rate in different surgical procedures

A total of four clinical studies with end-to-side or end-to-end anastomosis (9–12) and six clinical studies with longitudinal or triangular intussusception (13–18) were included in the subgroup analysis. In this subgroup analysis, the random-effect models were uniformly presented as a high inter-study heterogeneity (*P* = 0.32 *vs*. 0.0009, *I*^2^ = 14 vs. 76%, respectively). The results showed that the natural pregnancy rates for the vas deferens after end-to-side or end-to-end anastomosis and longitudinal or triangular intussusception were 20% and 23% (95% *CI* 15–26 vs. 13–34%, *P* < 0.0001 *vs. P* < 0.0001, Figure 4C), respectively.

#### 3.4.4. The ratio of pregnancy by ART in different surgical procedures

A total of four clinical studies with end-to-side or end-to-end anastomosis (9–12) and six clinical studies with longitudinal or triangular intussusception (13–18) were included in the subgroup analysis. In this subgroup analysis, the random-effect models were uniformly presented as a high inter-study heterogeneity (*P* = 0.01 *vs*. 0.0009, *I*^2^ = 73 vs. 76%), respectively. The results showed that the ratio of ART for the vas deferens after end-to-side or end-to-end anastomosis and longitudinal or triangular intussusception were 30% and 28% (95% *CI* 13–47 vs. 13–44%, *P* = 0.0005 *vs. P* = 0.0004, Figure 4D), respectively.

### 3.5. Publication bias test

**Figure 5:**
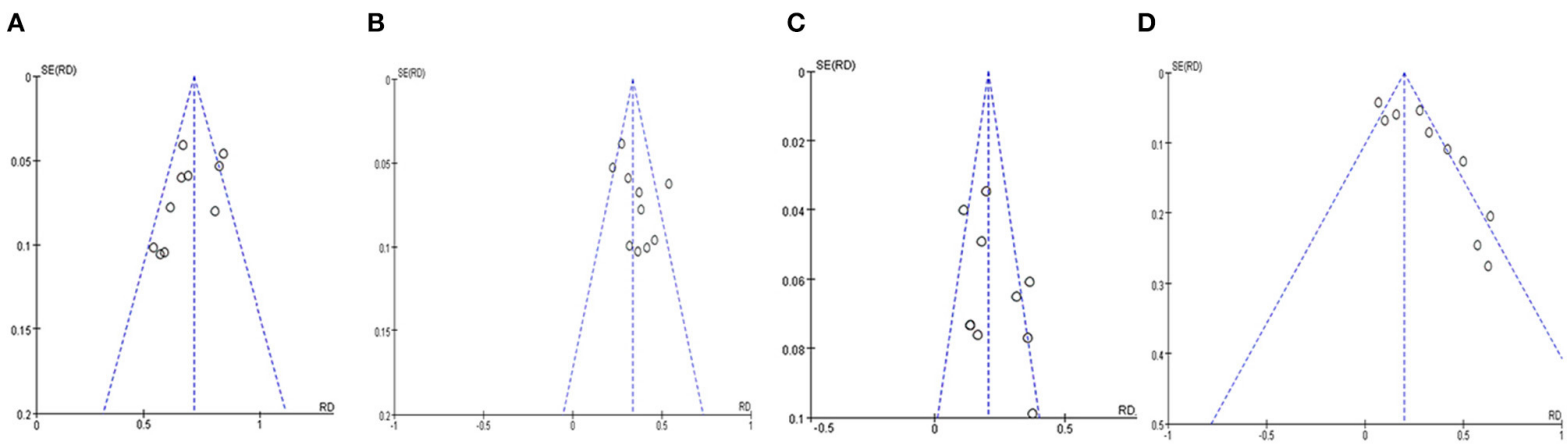
Funnel plots analysis for publication bias of pregnancy outcomes in patients with EOA after MVE. **(A)** Funnel plot of patency rate. **(B)** Funnel plot of overall pregnancy rate. **(C)** Funnel plot of natural pregnancy rate. **(D)** Funnel plot of the ratio of pregnancy by ART.

The funnel plots analysis of publication bias revealed general symmetry, and Begg's test results indicated that there was no strong evidence of publication bias (Figures 5A–D).

## 4. Discussion

Epididymal obstruction is the most common etiology of male infertility in patients with obstructive azoospermia (OA). Currently, assisted reproductive technology (ART), such as intracervical insemination (ICI), intrauterine insemination (IUI), *in vitro* fertilization and embryo transfer (IVF-ET), micro-testicular sperm extraction, and intracytoplasmic sperm injection (ICSI), has an obvious therapeutic effect on male infertility. However, there is still part of patients with epididymal obstructive azoospermia (EOA) (4) performing microsurgical vasoepididymostomy (MVE) to achieve natural pregnancy (19) and avoid ICSI complications, including ovarian hyperstimulation syndrome (OHSS) and multiple pregnancies (20). Single-center studies (21, 22) found a higher patency rate in male infertility patients with EOA after MVE (48.0%-71.7%), which is consistent with our meta-analysis (72%, 95% CI 68–76%). Therefore, the MVE procedure could obviously improve the overall patency rate of the vas deferens in male infertility patients with EOA.

However, the natural pregnancy rate in male infertility patients with EOA after MVE did not significantly improve. The results of our meta-analysis showed that the natural pregnancy rate is 21%, but the pregnancy success rate of ICSI is ~45–65% (6). Therefore, many male infertility patients with EOA require ART, including ICSI, to improve the pregnancy success rate of the spouse after the MVE procedure (8). Our meta-analysis included 54 patients who received ART after MVE to achieve a successful pregnancy in their spouses. The ratio of ART after MVE was 32.9%, and the pregnancy success rate of their spouses improved to 34%. Meanwhile, the application of ART could improve the chance of pregnancy regardless of postoperative sperm parameters. Therefore, we recommended that male infertility patients with EOA should receive ART after MVE to improve the rate of successful pregnancy in their spouses, including ICSI.

For patients with EOA after MVE, ICSI has become the preferred ART treatment strategy to improve fertilization rates (23), identify sperm morphology, and make up for the defect of lower sperm quality which failed to satisfy the demand for IUI and IVF-ET (24). Microsurgical epididymal sperm aspiration (MESA) has a higher rate of mate pregnancy than testicular sperm extraction (TESE). Therefore, MESA is the preferred epididymal sperm extraction procedure for male infertility patients with EOA after MVE (25).

Several abnormal complications during MVE, failure of vasectomy patency, and sperm dysfunction due to surgical trauma (5) have led EOA patients to receive micro-testicular sperm extraction and ICSI to achieve pregnancy after MVE, increasing the financial burden and secondary surgical trauma (24). The fertilization rates, spousal pregnancy rates, and abortion rates of cryopreserved sperms after ICSI were not significantly different compared with testicular fresh sperms (26, 27). The sperm motility after MVE was lower than the normal range according to World Health Organization (WHO) 6th guidelines in our systematic review. Therefore, we recommended that sperms from EOA male infertility patients should be cryopreserved in intraoperative MVE for application in the subsequent ICSI treatment procedure.

Previous studies found that the mean patency rate and mean pregnancy rates for end-to-side or end-to-end anastomosis were 61.1% and 29.9%, respectively. In contrast, the data on longitudinal or triangular intussusception developed to 69.1% and 36.9%, respectively (19). However, our meta-analysis found no significant differences in the overall patency rate (68 vs. 70%), the overall pregnancy rate (33 vs. 37%), the natural pregnancy rate (20 vs. 23%), and the ratio of ART (30 vs. 28%) among the different MVE procedures for end-to-side or end-to-end anastomosis and longitudinal or triangular intussusception. Therefore, changing the MVE procedure alone did not improve the pregnancy outcomes.

According to Binsaleh et al. (16), the average cost of MVE for patients in Western countries is more than $6000–9000. While the average cost of MVE in the Guangdong-Hong Kong-Macao Greater Bay Area of China is nearly ¥12000, and the financial burden of secondary micro-testicular sperm extraction combined with ICSI is similar to intraoperative cryopreserved sperm (¥43000–50000 *vs*. ¥42000–48000), with the same pregnancy rate of the spouse (55.2%). Therefore, there is no additional financial burden for patients with MESA cryopreserved human sperms in intraoperative MVE.

This study has the following limitations: first, for a lack of adequate data in these 10 clinical studies, we did not evaluate the effect of a spouse's gestational age on pregnancy outcomes. Previous studies have revealed that the optimal gestational age was lower than 39 years, and the overall pregnancy rate decreased with the age of the spouse (28), while there are significant differences between the average gestational age and non-pregnant age (26.5 *vs*. 32.7) (17). Therefore, further extensive searches are needed to investigate the gestational age of the spouses. Second, we did not evaluate the pregnancy rate of those who received ART after MVE. Since the included literature did not contain all the information on ART after MVE, we performed a meta-analysis only on the ratio of patients with pregnancy by ART after MVE for these patients who achieved the final outcome of pregnancy. Therefore, more extensive systematic studies are needed to evaluate the pregnancy rate of ART after MVE. Next, for the lack of these patients' data from adolescence, we cannot evaluate the correlation between the duration of obstruction and pregnancy outcomes. Finally, due to the lack of a randomized controlled study (RCT), we conducted only single-group studies on pregnancy outcomes. Therefore, we planned to continue prospective or retrospective randomized controlled clinical studies on this topic.

## 5. Conclusion

Vasectomy patency rates are higher, but natural pregnancy rates are lower in EOA male infertility patients after MVE. Altering the MVE procedures alone does not significantly improve pregnancy outcomes, but ART after MVE could improve the chance of pregnancy regardless of sperm parameters. We recommended that human sperms from EOA male infertility patients should be cryopreserved during intraoperative MVE for application in the subsequent ICSI treatment procedure.

## Data availability statement

The original contributions presented in the study are included in the article/Supplementary material, further inquiries can be directed to the corresponding author.

## Author contributions

XX and ZW contributed to the design and conception of the study, and the acquisition of data was performed by XW, CS, FL, and JZ. Analysis and interpretation of data were performed by ZW, XW, NL, and BJ. The manuscript was drafted by ZW, XW, and ST. The critical revision of the manuscript was performed by CS, FL, and ST. All authors approved the submitted and final versions.
