# Supplementary material for: The pregnancy outcomes in patients with epididymal obstructive azoospermia after microsurgical vasoepididymostomy: a systematic review and meta-analysis
Source: Front Med (Lausanne). 2023 May 18;10:1186729. doi: 10.3389/fmed.2023.1186729 (PMC10233013; doi:10.3389/fmed.2023.1186729)
Supplement: Supplementary file 1 [file Image_1.PDF]

A

Study or Subgroup	Overall pregnancy Mean	SD	Total	Unpregnancy Mean	SD	Total	Weight	Mean Difference IV, Random, 95% CI
Binsaleh at al 2014	29.7	30.7	22	17.6	30.71	14	13.2%	12.10 [-8.48, 32.68]
Hibi et al 2000	7.7	12.16	24	1	12.16	13	83.1%	6.70 [-1.51, 14.91]
Matsuda et al 1994	44.5	57.66	24	23.2	60.07	14	3.7%	21.30 [-17.72, 60.32]
**Total (95% CI)**			**70**			**41**	**100.0%**	**7.95 [0.47, 15.43]**

Heterogeneity: Tau² = 0.00; Chi² = 0.70, df = 2 (P = 0.71); I² = 0%
Test for overall effect: Z = 2.08 (P = 0.04)

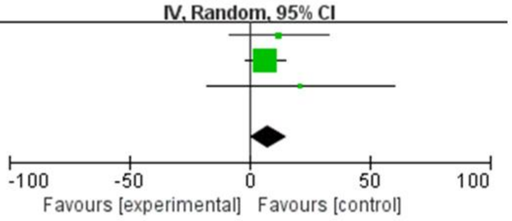

B

Study or Subgroup	Overall pregnancy Mean	SD	Total	Unpregnancy Mean	SD	Total	Weight	Mean Difference IV, Random, 95% CI
Binsaleh at al 2014	11	10.42	22	7.3	10.42	14	49.1%	3.70 [-3.28, 10.68]
Hibi et al 2000	9.6	13.11	24	2.9	13.11	13	38.4%	6.70 [-2.15, 15.55]
Matsuda et al 1994	29.2	28.91	24	7.4	28.5	14	12.6%	21.80 [2.91, 40.69]
**Total (95% CI)**			**70**			**41**	**100.0%**	**7.12 [-0.09, 14.33]**

Heterogeneity: Tau² = 14.88; Chi² = 3.13, df = 2 (P = 0.21); I² = 36%
Test for overall effect: Z = 1.94 (P = 0.05)

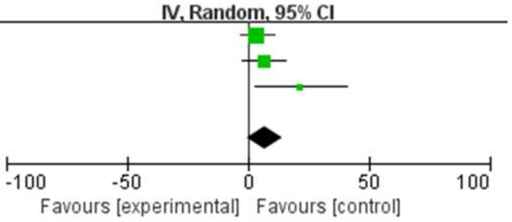

C

Study or Subgroup	Natrual pregnancy Mean	SD	Total	Unpregnancy Mean	SD	Total	Weight	Mean Difference IV, Random, 95% CI
Binsaleh at al 2014	28.8	30.7	17	17.6	30.71	14	13.2%	11.20 [-10.52, 32.92]
Hibi et al 2000	7.2	12.16	18	1	12.16	13	82.8%	6.20 [-2.47, 14.87]
Matsuda et al 1994	46	57.66	23	23.2	60.07	14	4.0%	22.80 [-16.51, 62.11]
**Total (95% CI)**			**58**			**41**	**100.0%**	**7.53 [-0.36, 15.42]**

Heterogeneity: Tau² = 0.00; Chi² = 0.78, df = 2 (P = 0.68); I² = 0%
Test for overall effect: Z = 1.87 (P = 0.06)

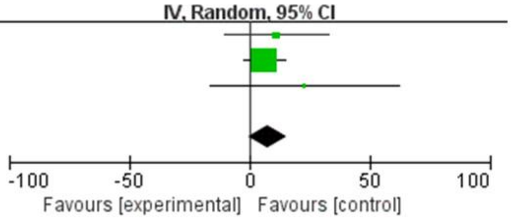

D

Study or Subgroup	Natural pregnancy Mean	SD	Total	Unpregnancy Mean	SD	Total	Weight	Mean Difference IV, Random, 95% CI
Binsaleh at al 2014	10.5	10.42	17	7.3	10.42	14	49.4%	3.20 [-4.17, 10.57]
Hibi et al 2000	9.1	13.11	18	2.9	13.11	13	37.8%	6.20 [-3.15, 15.55]
Matsuda et al 1994	28.5	28.91	23	7.4	28.5	14	12.8%	21.10 [2.06, 40.14]
**Total (95% CI)**			**58**			**41**	**100.0%**	**6.63 [-0.68, 13.94]**

Heterogeneity: Tau² = 14.00; Chi² = 2.97, df = 2 (P = 0.23); I² = 33%
Test for overall effect: Z = 1.78 (P = 0.08)

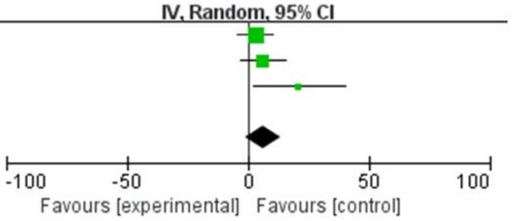

E

Study or Subgroup	Pregnancy by ART Mean	SD	Total	Unpregnancy Mean	SD	Total	Weight	Mean Difference IV, Random, 95% CI
Binsaleh at al 2014	32.8	31.3	5	17.6	30.71	14	14.5%	15.20 [-16.60, 47.00]
Hibi et al 2000	8.9	14.14	6	1	12.16	13	85.5%	7.90 [-5.20, 21.00]
Matsuda et al 1994	9.4	0	1	23.2	60.07	14		Not estimable
**Total (95% CI)**			**12**			**41**	**100.0%**	**8.96 [-3.16, 21.07]**

Heterogeneity: Tau² = 0.00; Chi² = 0.17, df = 1 (P = 0.68); I² = 0%
Test for overall effect: Z = 1.45 (P = 0.15)

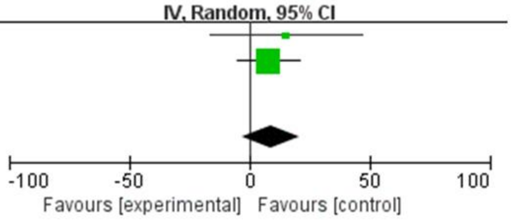

F

Study or Subgroup	Pregnancy by ART Mean	SD	Total	Unpregnancy Mean	SD	Total	Weight	Mean Difference IV, Random, 95% CI
Binsaleh at al 2014	12.8	10.09	5	7.3	10.42	14	63.0%	5.50 [-4.89, 15.89]
Hibi et al 2000	11.2	14.44	6	2.9	13.11	13	37.0%	8.30 [-5.28, 21.88]
Matsuda et al 1994	46	0	1	7.4	28.5	14		Not estimable
**Total (95% CI)**			**12**			**41**	**100.0%**	**6.53 [-1.72, 14.79]**

Heterogeneity: Tau² = 0.00; Chi² = 0.10, df = 1 (P = 0.75); I² = 0%
Test for overall effect: Z = 1.55 (P = 0.12)

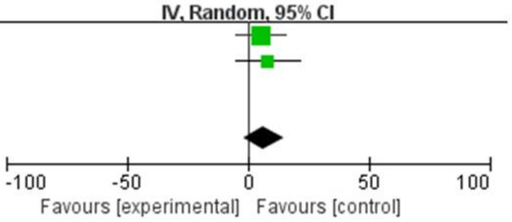

Supplementary figure 1 Continuous meta-analysis between pregnancy outcomes and sperm parameters in patients with EOA after MVE. (A,B) Meta-analysis of overall pregnancy with sperm count (A) or sperm motility (B). (C,D) Meta-analysis of natural pregnancy with sperm count (C) or sperm motility (D). (E,F) Meta-analysis of patients with pregnancy by ART with sperm count (E) or sperm motility (F).
